# Associations of Perceived School and Year Group Climate with Mental Health Among Children Aged 7-to-11 Years

**DOI:** 10.1007/s12187-024-10213-7

**Published:** 2025-01-08

**Authors:** Caitlyn Donaldson, Kelly Morgan, Safia Ouerghi, James J. Lewis, Graham Moore

**Affiliations:** 1 Centre for Development, Evaluation, Complexity and Implementation in Public Health Improvement (DECIPHer), School of Social Sciences, Cardiff University, Sbarc, Maindy Road, Cardiff, CF24 4HQ UK; 2 Wolfson Centre for Young People’s Mental Health, Cardiff University, Cardiff, UK

**Keywords:** Mental health, Children, Schools, Intra-class Correlation Coefficients (ICCs), School climate

## Abstract

Schools are an important setting for interventions to improve mental health. There is growing evidence that school climate – sometimes expressed as the perceptions that children have about the relationships, safety, values, and beliefs within their school – can impact child mental health. Poor child mental health is associated with feelings of distress as well deficits in functioning. However, while most studies have focused on school climate, climate at lower levels of nesting, including year group, may be important. Cross-sectional data on emotional and behavioural difficulties from 32,606 children in primary schools in Wales (ages 7–11, year groups 3–6) were collected via a school survey, delivered online and within the classroom environment to all children who consented, and analysed using multilevel modelling. Models were then extended to consider how aggregated measures of year group and school climate are associated with mental health outcomes. The unadjusted variance partition coefficients (VPCs) indicated that 2.8% of the variance in emotional difficulties and 3.2% in behavioural difficulties were attributable to differences in the year group, while school-level differences represented 2.4% and 3.5%, respectively. More positive year group and school climate were associated with more positive mental health outcomes. School and year group climate are independently associated with primary school children’s mental health. Interventions to support mental health should aim to optimise the whole school climate, as well as climate within year group clusters.

**Supplementary Information:**

The online version contains supplementary material available at 10.1007/s12187-024-10213-7.

## Introduction

Schools are important settings for intervention as they offer access to large numbers of children within a relatively controlled environment. The use of schools as intervention settings also reflects an assumption that schools, through their practices, have an impact on children’s outcomes. Interventions to support children’s mental health are common within schools (e.g. Caldwell et al., 2019; O’Reilly et al., 2018) and may focus on a range of different outcomes including depression, anxiety, mental and emotional wellbeing, as well as behavioural outcomes (Caldwell et al., 2019; Clarke et al., 2021; Mackenzie & Williams, 2018; Mendes de Oliveira et al., 2022; Zhang et al., 2023). Interventions may target specific groups or be universal, aiming to shift outcomes in a favourable direction for all children (Rose, 2001). There is particular evidence of effectiveness for whole school intervention approaches for mental health in schools (Bonell et al., 2018; Goldberg et al., 2019; Shinde et al., 2018). They seek to influence the relationship between policies, practices and relationships at a school level, and children’s outcomes, and in doing so implicitly acknowledge the nested nature of school systems.

One key area of focus for intervention research is on school climate, which reflects perceptions of school social interactions, relationships, safety and values, by children, teachers, and other school staff (Rudasill et al., 2018). The INCLUSIVE trial, for example, showed that experimentally improving school climate does lead to small improvements in child mental health within secondary schools (Bonell et al., 2018).

However, a focus on school-level effects may overlook how nesting at lower levels within the school, including within year groups, might also create variation in effects. Children are nested in year groups based on age, and therefore represent a group of children at a similar developmental stage, with similar school experiences in terms of curriculum, pedagogy and disciplinary practices, which may differ to children in other years of the school. Pedagogy, for example, is aligned to the educational needs of the children, and will involve scaffolding based on level of development and previous knowledge (Bliss et al., 1996; Peterson et al., 2018). Acceptable behaviour and levels of responsibility will also be based on the developmental stage of the child, and the same behaviour may be perceived differently based on the size and age of the child, resulting in different disciplinary responses (Welsh, 2022). The perceptions the young people have of relationships, safety, values, and beliefs may therefore also vary from children in other school years.

Class climate, where most research on lower levels of nesting in schools has concentrated (e.g. Wang et al., 2020), is likely to differ from year group climate where schools have multiple classes in a year group, but converge when there is one class per year group. In the case of multiple classes in a year group, classroom effects will differ within year groups due to different teachers and other staff members (for example, teaching assistants) (Kirabo Jackson et al., 2014), a different physical environment due to separate classrooms, and also differences in the characteristics of the children that make up each class. However, focusing on class climate may overlook age effects when there is mixing of year groups within a class. Focusing on year group climate allows us to consider how developmental stage within the context of a specific school, might be related to school climate and mental health. This is particularly relevant when considering how interventions might be targeted at a whole year group level.

### Contextual and Composition Factors

School and year group effects on health may be due to both contextual and compositional factors (Bonell et al., 2013). Both of these factors will have an effect on the clustering of young people’s mental health.

Compositional factors refer to the children and their individual characteristics suggesting that it is factors such as the socioeconomic status (SES) or the ethnicity of the children that are responsible for clustering of health outcomes. In terms of year groups, age is an important compositional factor, that may, irrespective of the school environment, influence child mental health outcomes, and thus create a clustering effect. Our recent analysis found evidence of an increase in both emotional and behavioural difficulties with increased age in primary school children (Donaldson et al., 2023).

Contextual arguments suggest that the characteristics of the school itself create a clustering effect – for example, the shared physical environment, school policies and priorities, ethos and culture. From a contextual factor perspective, Bernstein (1975) proposes that school culture is an important predictor of health outcomes, and that it is expressed as two different orders – the regulatory and instructional orders. The former sets the expected rules and relational aspects of the school culture, while the latter sets the academic expectations. Through their influence on how children build the resources of practical reasoning and affiliation, the two cultural orders indirectly impact upon child health (Markham & Aveyard, 2003). However, while culture is primarily discussed at the school-level, it is feasible that the regulatory and instructional orders may also operate at the year group level. Children of different ages are likely to have different expectations and rules in terms of both behaviour and academics placed upon them (Bliss et al., 1996; Peterson et al., 2018; Welsh, 2022). This has the potential to create sub-cultures of the regulatory and instructional orders, based on the developmental needs of children of different ages.

It is the child’s perceptions of the regulatory and instructional orders through their influence on different aspects of the school environment that produces a measure of school climate. Rudasill et al. (2018) suggest that while compositional effects of the school based on the characteristics of its members can affect the development of perceptions (for example, age in the case of year group effects) it is the group-level perceptions themselves that reflect school and year group climate. However, while theoretically age as a compositional factor, and year group as a contextual factor are different, in practice, within the Welsh educational system, age and year group are so closely aligned that separating out the relative effect of each is very difficult.

### Year Group and School Climate

Differences in the conceptualisation and measurement of school and year group climate can make unpicking their relationship with child mental health complicated. As there is no gold standard tool to assess school climate, studies often develop new measures rather than using those already validated (Grazia & Molinari, 2021). However, a review of child-reported measures of school climate found that despite the confusion, researchers were mostly “hunting the same beast” (Lewno-Dumdie et al., 2020, p.15), and that most child-report measures of school climate included aspects of relationships, safety and institutional environment. Rudasill et al. (2018) argues that the school should be viewed as a microsystem within which are a number of nested nanosystems (Bronfenbrenner, 1979). This allows year group climate to be seen as separate to, and yet closely related to overall school climate.

There has been no previous research on year group climate and child mental health, but a number of studies have sought to better understand the relationship between school climate, class climate and mental health. The findings are generally consistent: more positive class and school climate tends to be associated with better child mental health. For example, in a meta-analysis, with class climate composed of instructional, socioemotional and organisational class processes, the authors found evidence that higher class climate was associated with lower externalising behaviour and socioemotional distress (Wang et al., 2020). At the school-level, Wang and Degol (2016) reviewed evidence on climate and mental health and found that measures including school belonging, relationships and safety were important predictors of emotional wellbeing. A systematic review by Aldridge and McChesney (2018) considered evidence of mental health outcomes for a number of school climate dimensions in secondary-aged children, finding that positive perceptions of relationships, school safety and school connectedness were all associated with more positive mental health outcomes – while a high demand academic climate tended to be associated with more negative mental health outcomes.

Most of the studies included in these three reviews (Aldridge & McChesney, 2018; Wang & Degol, 2016; Wang et al., 2020), were cross-sectional and therefore limited in assessing causality between climate and mental health. However, Raniti et al. (2022)’s systematic review focused on longitudinal and intervention studies. The authors found evidence that higher levels of school connectedness (the review included ‘school belonging’ and ‘school climate’ within the definition of school connectedness) predicted lower levels of depressive and/or anxiety symptoms at a later point. On a similar theme, analysis of longitudinal data from secondary-aged children before and during the COVID-19 pandemic also found evidence of a causal relationship between school climate and mental health. While children with more positive perceptions of school climate before the pandemic had lower levels of depression and higher positive affect during the pandemic compared to their peers, they also saw steeper negative changes in these outcomes during the pandemic, suggesting that these children relied more heavily on school to support their mental health and were more adversely affected by school closures and other school disruptions (Chen et al., 2024). These studies provide evidence of a causal link between school climate and child mental health outcomes, which reflects theoretical arguments that where schools are supportive of young people’s academic and emotional development, they can develop practical reasoning and affiliation, resources required for making positive decisions that impact upon health (Markham & Aveyard, 2003). However, the relationship is likely to operate in the reverse direction too, as schools with high numbers of children with poor mental health (possibly due to compositional factors, such as deprivation) may have lower perceptions of the school climate as a result.

### Measuring Clustering of Mental Health Outcomes

**Fig. 1:**
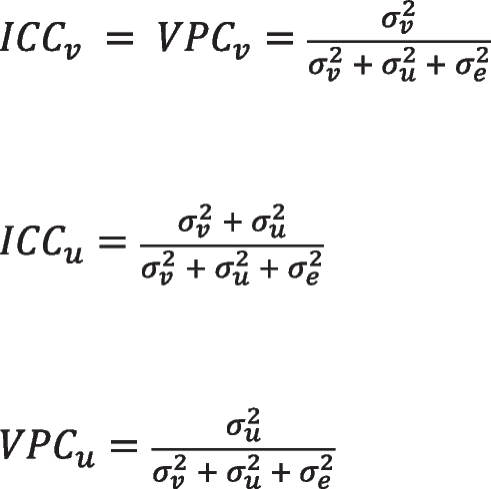
Formulae for calculating ICCs and VPCs from variance components at each level of the model. v = school level; u = year group level; e = individual level. In all cases, within a logit model, individual variance is estimated to be π^2^/3 (Steele, 2009)

The strength of a ‘nesting’ or ‘clustering’ effect is typically assessed using an intraclass correlation coefficient (ICC). In three level models – children nested within year groups and year groups nested within schools, the ICC at the school level is the correlation between two children for a given outcome within the same school, but different classrooms. The ICC at the class level is the correlation between two children within the same year group and also therefore the same school (Leckie, 2013). An alternative approach to assessing variance components within multilevel models is the variance partition coefficient (VPC). This provides the percentage of variance associated with each level of the model. In a simple three-level model (children in year groups in schools), the VPC at the school level represents the proportion of variance attributed to systematic differences between schools (and is the same value as the school-ICC). However, at the year group-level, the VPC diverges from the ICC, and represents the proportion of variance that lies within schools, between year groups (Fig. 1). There is no corresponding ICC interpretation of the year group VPC because it implies a correlation between children in different schools but the same year group cluster, which is not possible within nested data (Leckie et al., 2020). Both statistics are useful in understanding where variability lies within the multi-level model and its relative importance.

In schools, ICCs for educational outcomes are typically higher than for health outcomes. US data suggest average unadjusted ICCs for children from kindergarten to grade 12 of 0.22 for both mathematics and reading (Hedges & Hedberg, 2007). Similarly, attainment data from English secondary schools found unadjusted ICCs of 0.19–0.25 based on data from two datasets. This contrasts with unadjusted ICCs for behavioural difficulties which ranged from 0.01–0.04 and mental and emotional health ICCs which ranged from 0.00–0.06 depending on child age, dataset used and outcome measure (Hale et al., 2014). Shackleton et al. (2016) explored ICCs in European data from children aged 15–16 years old. For depressive mood, unadjusted ICCs ranged from 0.01–0.07 in data from 11 countries (ICC from Britain was 0.03), and for self-esteem ICCs ranged from 0.01–0.08 in data from nine countries (ICC from Britain was 0.01).

A recent systematic review of health-related ICCs from school-based cluster randomised controlled trials found 39 studies that assessed school ICCs for socioemotional functioning and its influences (which included a broad range of outcomes including mental health, behaviour, neurodiversity, wellbeing, bullying and self-esteem). The median unadjusted ICC was 0.05 with a range of 0.00 to 0.22 (Parker et al., 2023). Four studies reported unadjusted class-level ICCs alongside unadjusted school ICCs for socioemotional functioning and its influences. Three of these only included primary-aged children: cyberbullying (0.04 for school; 0.09 for class) (Williford et al., 2013); time on task (0.09 for school and 0.14 for class) (Bartholomew et al., 2018) and social skills (0.08 for school and 0.18 for class) (DiPerna et al., 2015). These papers suggest that children within the same classrooms tend to be more alike for these outcomes than children in different classrooms within the same school – however, ICCs remain relatively low at both class and school level relative to academic benchmarks. The implication is that either schools have little impact on child mental health because school level effects describe a very small proportion of the overall variability, or that schools have a larger effect than is reflected in the ICCs because they are relatively homogenous in their effects, and therefore children in different schools are more alike than anticipated. In terms of year groups, it suggests that, by extrapolating the evidence from class-level ICCs, it is likely that children within year groups will be more alike than those in different year groups, not just because they are of the same age, but also because there is a shared environment.

### Research Questions

This research will seek to fill a number of research gaps. Firstly, it is not clear whether year group clustering should be modelled within primary school data alongside school level clustering, and the relative size of each effect. Secondary, there is a gap in understanding the extent to which year group and school climate might operate independently to shape clustering of mental health outcomes. Clustering at the year group level may be replicating school-level effects or acting independently. The calculation of school-level variance is based on the difference between mean values for each school and an overall mean, while year group variance is based on the difference between year group mean values and its specific school mean. It means that while there may be low between school variation because on average school mean values are quite similar, this may mask variation at the year group level, which may display opposing trends. This is important because if year group climate effects are independent or additive to school climate effects, it is important to consider how schools can ensure that each year group nanosystem is supportive of mental health, rather than solely considering the school microsystem as a whole. Using cross-sectional data, this study will therefore seek to explore:

- RQ1: To what extent do emotional and behavioural difficulties differ by school, and year group within school, among primary school children in Wales?
- RQ2: Are individual perceptions of school, and aggregated child perceptions of year group and school climate, independently associated with emotional and behavioural difficulties?

## Methods

In September 2022-March 2023, 32,606 primary school children from 354 schools in Wales completed an online Student Health and Well-being survey as part of a feasibility trial run by the School Health Research Network (SHRN). Among schools, an additional 628 children opened the survey, but declined to participate (1.9%). Consent was based on a three tiered opt-out procedure. Initial consent was received from schools by returning a completed research agreement. Schools provided parents/carers with a participant information sheet providing an overview of the study, and a copy of the questionnaire. Parents/carers were given 14 days to opt out their child from participating by notifying the school. Alternative tasks were provided for these students on the day of data collection. Children were informed about the survey in advance and on the day of data collection, were given the option to decline participation. Children could also skip questions or stop completing the survey at any time. No data are available on children who partially completed the survey, opted out of participating, or were away from school the day of the survey in order to calculate an overall response rate. However, based on school census data, the sample (*n* = 32,606) represents 23% of children in years 3–6 in Wales (Donaldson et al., 2023). School-level percentages of children eligible for free school meals were close to the national average (21.6% in this sample, compared to 20.1% nationally). There was some variation based on regional participation: the South East Wales region had the highest proportion of schools participating (40.4%), and the North Wales region had the lowest (21.1%). However, data weighed by local authority produced estimates very close to unweighted values (see Appendix 1), suggesting the data are representative of the overall population.

### Measures

#### Mental Health

The Me and My Feelings questionnaire (Deighton et al., 2013) is a 16-item measure that assesses emotional (10 items) and behavioural difficulties (six items). It has been validated for use in children from age eight in UK community samples (Patalay et al., 2014).

All items are rated on a scale of 0–2, where 0 = never, 1 = sometimes and 2 = always. Items for emotional difficulties include, ‘I feel lonely’; ‘I feel scared’; ‘I worry when I am at school’ and ‘I cry a lot’. The scale is scored out of 20. Scores of 10 and higher indicate elevated or clinically significant emotional difficulties (28% of children), and were used to create a binary outcome measure. For behavioural difficulties, items include, ‘I get very angry’; ‘I do things to hurt people’; ‘I break things on purpose’ and ‘I hit out when I am angry’. The scale is scored out of 12. Scores of six and higher indicate elevated or clinically significant behavioural difficulties (14% of children), and were used to create a binary outcome measure. Scores were ‘pro-rated’ (i.e. based on the average of completed items) so long as > 50% of individual items were completed for each subscale.

#### Demographic Covariates

As there was no direct measure of age for students in the dataset, school year was used as a proxy (years 3–6, corresponding to ages 7 to 11 years). As it is rare for children not to be of the ‘correct’ age in a school year in the UK, this acts as a good proxy. School year (years 3–6) was also used as a clustering variable (from here on, the term ‘school year’ in this paper refers to the variable being used as at the individual-level, while ‘year group’ refers to it within the context of higher level clustering). Aligning the survey data to data from Stats Wales (Stats Wales, 2024), suggests that half (50.6%) of participating schools had eight or fewer classes (which would equate to one class or fewer per year from nursery to year 6) and very few schools had 16 or more classes – i.e. two classes or more in all school years (15.2%) (see Appendix 2).

Gender was assessed through a question that asked children whether they were a ‘boy’, ‘girl’ or ‘neither word describes me’. Family structure asked children which adults they live with all or most of the time and was dichotomised for the analysis into ‘both parents’ and ‘other family structure’.

#### Individual, Year Group and School Climate

Three items were used to create measures of individual, year group and school-level perceptions of climate. These were the extent to which children feel that their teachers care about them, the extent to which they feel that they belong at school, and the extent to which they like school (full questions are in Appendix 3). These items were selected as they were asked of all year groups (additional questions used to assess school climate in other analyses of this survey were asked of older year groups only). ‘Teachers care’ and ‘school belonging’ were scored on a five point likert scale, while ‘liking school’ was scored on a four point likert scale, with higher scores indicating more positive perceptions. At the individual-level, items were not strongly correlated and alpha was low (Appendix 4), therefore items were included within the models separately. At the year and school level, a mean value for each item was calculated. These mean values were summed at each level to create two climate measures which had good internal consistency (alpha for year group climate = 0.83; alpha for school climate = 0.86).

### Missing Data

There was a small amount of missing data in the outcome variables (*n* = 719, 2.2% for emotional difficulties; *n* = 891, 2.7% for behavioural difficulties) and other predictor variables (Appendix 5). Univariate analysis using chi squared tests was used to determine which variables should be used for multiple imputation (Appendix 6). All variables selected were significantly associated with the outcome variables, except for survey language and behavioural difficulties (*p* = 0.663). Missingness in emotional difficulties was significantly associated with lower school year, being in a less affluent school, have more negative perceptions about school, having low levels of life satisfaction, and doing higher levels of exercise (details on these questions are in Appendix 6). Missingness in behavioural difficulties was significantly associated with lower school year, not living with two parents, more negative perceptions about school, lower life satisfaction, higher levels of exercise and being a recent victim of bullying. These variables were included within the imputation models.

### Analysis

Data were cleaned in Stata 17.0 and then transferred to R (version 4.3.2) for multiple imputation. The mice and miceadds packages were used to impute variables while accounting for the three-level multilevel structure of the data (Grund, 2019; Wijesuriya et al., 2020). Climate measures at the year group and school level were aggregated after imputation to ensure that imputed values were included within the calculations – if aggregated before imputation there was no missing data at the aggregate level to impute. Twenty imputations, each with 20 iterations, were run and then transferred back to Stata 17.0 for analysis. Four sensitivity analyses were carried out (Appendices 7–16) and suggest no substantive departure from the main analysis model.

Multi-level logistic regression with emotional difficulties and behavioural difficulties as outcomes was carried out using Stata 17.0 and ICCs and VPCs calculated for each model. As likelihood ratio tests (LRTs) require maximum likelihood estimates which are not produced when analysing multiply imputed data, LRTs were run on the first imputation to provide approximate values and enable models to be compared. Three level substantive models were compared to two alternative two-level substantive models – children in year group (no school-level effect) and children in schools (no year group-level effect), in order to provide justification for use of three-level models for the later analysis.

A number of three level models were then run. Model 1 was adjusted for gender, school year and family structure. Model 2 added the three individual-level school climate measures. Models 3 and 4 built on Model 2 to add year group and school climate measures respectively. Finally Model 5 was the full model with all predictors included. Models 1–4 were compared to Model 5 using likelihood ratio tests on the first imputation.

## Results

Descriptive statistics at the individual-level are presented in Table 1 based on pre-imputed data. The proportion of children who responded was evenly split between boys (*n* = 15,685; 49.1%) and girls (*n* = 15,730; 49.2%), with a small number of children with a different gender identity (*n* = 559; 1.8%) participating. Most children (*n* = 23,633; 76.9%) lived with both parents. The numbers of children who participated increased with age (22.9% were in year 3; 23.9% in year 4; 25.3% in year 5; and 28.0% were in year 6). There were 354 schools and 1340 year group clusters (323 in year 3; 331 in year 4; 341 in year 5; 345 in year 6). For year group climate, the aggregated mean score was 11.8 (range 7.0–14.0), and 11.8 (range 9.9–13.1) for school climate.

**Table 1:** Descriptive statistics for the sample

	*N* (%)
Gender	
Boy	15,685 (49.1%)
Girl	15,730 (49.2%)
Other gender identity	559 (1.8%)
School year/age	
Year 3 (age 7–8)	7,457 (22.9%)
Year 4 (age 8–9)	7,780 (23.9%)
Year 5 (age 9–10)	8,251 (25.3%)
Year 6 (age 10–11)	9,118 (28.0%)
Family structure	
Live with both parents	23,633 (76.9%)
Single mother	4,393 (14.3%)
Step family	1,570 (5.1%)
Single father	438 (1.4%)
Grandparents	272 (0.9%)
Foster family	236 (0.8%)
Other family structure	174 (0.6%)

Unadjusted and adjusted ICCs for emotional and behavioural difficulties are presented in Table 2. Two level models of children within schools found unadjusted ICCs of 0.031 for emotional difficulties and 0.043 for behavioural difficulties, suggesting that approximately 3–4% of variance in mental health difficulties was between schools rather than individuals. Two-level models of children within year groups found unadjusted ICCs of 0.051 for emotional difficulties and 0.065 for behavioural difficulties.

**Table 2:** Intraclass correlations for emotional and behavioural difficulties (unadjusted and adjusted for all individual-level variables) (*n* = 32,606)

	Three levels (children in year groups in schools)		Two level (children in year groups)		Two level (children in schools)	
	Year group ICC (95%CI) VPC	School ICC (95%CI) VPC	Yeargroup ICC (95%CI) VPC	Likelihood ratio test (compared to three level model)	School ICC (95%CI) VPC	Likelihood ratio test (compared to three level model)
Emotional difficulties unadjusted	0.052 (0.042–0.061) 2.81%	0.024 (0.016–0.032) 2.37%	0.051 (0.042–0.060) 5.08%	67.57 (*p* < 0.001)	0.031 (0.023–0.039) 3.10%	80.91 (*p* < 0.001)
Emotional difficulties adjusted (school year)	0.050 (0.041–0.060) 2.60%	0.024 (0.016–0.032) 2.44%	0.049 (0.040–0.058) 4.91%	72.62 (*p* < 0.001)	0.031 (0.024–0.039) 3.11%	70.28 (*p* < 0.001)
Emotional difficulties adjusted (school year, gender, family structure)	0.049 (0.040–0.059) 2.58%	0.023 (0.016–0.031) 2.34%	0.048 (0.039–0.057) 4.79%	66.78 (*p* < 0.001)	0.030 (0.023–0.038) 3.02%	68.20 (*p* < 0.001)
Behavioural difficulties unadjusted	0.067 (0.053–0.080) 3.20%	0.035 (0.023–0.046) 3.47%	0.065 (0.052–0.078) 6.53%	63.57 (*p* < 0.001)	0.043 (0.032–0.054) 4.31%	47.39 (*p* < 0.001)
Behavioural difficulties adjusted (school year)	0.064 (0.051–0.078) 2.87%	0.036 (0.024–0.047) 3.57%	0.063 (0.050–0.076) 6.31%	68.91 (*p* < 0.001)	0.043 (0.032–0.054) 4.34%	38.93 (*p* < 0.001)
Behavioural difficulties adjusted (school year, gender, family structure)	0.061 (0.047–0.074) 2.85%	0.032 (0.021–0.043) 3.23%	0.059 (0.047–0.072) 5.93%	58.74 (*p* < 0.001)	0.040 (0.029–0.050) 3.98%	36.79 (*p* < 0.001)

Likelihood ratio test statistics based on first imputation. Complete case statistics available in supplementary material

*ICC* Intra-class correlation, *VPC* variance partition coefficient

^*^Significant p-values indicate that the reduced model was a significantly worse fit than the three-level model

^**^All two-level models were a significantly better fit to the data than a one-level model with no clustering effect (*p* < 0.001 for all models)

In three-level models, the unadjusted ICC for emotional difficulties at the year group level was 0.052 and at the school level was 0.024. VPCs for emotional difficulties suggest that 2.4% (reflecting the ICC) lies between schools, 2.8% lies within schools between year groups and 94.8% lies within year groups between children. For behavioural difficulties the unadjusted ICC at the year group level was 0.067 and at the school level was 0.035. 3.5% of the variability lies between schools, 3.2% lies within schools between year groups and 93.3% lies within year groups between children.

For the unadjusted models, the three level models were a significantly better fit for both emotional difficulties (chi2 = 67.6, *p* < 0.001 compared to children in year groups; chi2 = 80.9, *p* < 0.001 compared to children in schools) and behavioural difficulties (chi2 = 63.6, *p* < 0.001 compared to children in year groups; chi2 = 47.4, *p* < 0.001 compared to children in schools). All adjusted models were also a significantly better fit when three-levels were modelled compared to two-levels (*p* < 0.001 for all comparisons). These findings suggest that mental health difficulties cluster at both the school and year group level among primary school in Wales (RQ1).

After models were adjusting for school year (proxy for child age), ICCs at the year group level only marginally decreased (0.052 to 0.050 for emotional difficulties; 0.067 to 0.064 for behavioural difficulties). This suggests that while age is having some impact on the clustering of outcomes, there remains variability at the year group level that is not explained by age similarities between children.

Tables 3 and 4 present the logistic regression parameters alongside ICCs, VPCs and likelihood ratio tests for emotional and behavioural difficulties.

**Table 3:** Multi-level logistic regression (emotional difficulties) (*n* = 32,606)

	Model 1: Three level, no individual perceptions; no year group or school climate (OR, 95%CI)	Model 2: Three level with no year group or school climate (OR, 95%CI)	Model 3: Three level with year group climate only (OR, 95%CI)	Model 4: Three level with school climate only (OR, 95%CI)	Model 5 (Full model): Three level with year group and school climate (OR, 95%CI)
School year	1.07 (1.04–1.10) *p* < 0.001	0.98 (0.96–1.01) *p* = 0.284	0.96 (0.93–0.99) *p* = 0.004	0.99 (0.96–1.01) *p* = 0.287	0.96 (0.93–0.99) *p* = 0.020
Teachers care	-	0.87 (0.84–0.91) *p* < 0.001	0.88 (0.85–0.92) *p* < 0.001	0.88 (0.84–0.91) *p* < 0.001	0.88 (0.85–0.92) *p* < 0.001
Belong	-	0.68 (0.66–0.71) *p* < 0.001	0.69 (0.67–0.71) *p* < 0.001	0.69 (0.66–0.71) *p* < 0.001	0.69 (0.67–0.71) *p* < 0.001
Like school	-	0.73 (0.70–0.75) *p* < 0.001	0.73 (0.71–0.76) *p* < 0.001	0.73 (0.71–0.76) *p* < 0.001	0.73 (0.71–0.76) *p* < 0.001
Year group climate	-	-	0.89 (0.84–0.93) *p* < 0.001	-	0.91 (0.86–0.97) *p* = 0.004
School climate	-	-	-	0.84 (0.78–0.92) *p* < 0.001	0.92 (0.83–1.01) *p* = 0.092
Year group ICC (95%CI)	0.049 (0.040–0.059)	0.036 (0.027–0.045)	0.034 (0.025–0.042)	0.034 (0.026–0.043)	0.034 (0.025–0.042)
School ICC (95%CI)	0.023 (0.016–0.031)	0.017 (0.010–0.024)	0.015 (0.009–0.022)	0.015 (0.008–0.021)	0.015 (0.009–0.022)
Year group/ School VPC	2.58%/2.34%	1.91%/1.69%	1.85%/1.54%	1.93%/1.49%	1.85%/1.51%
Likelihood ratio test (compared to Model 5—full model)	2432.43 (*p* < 0.001)	25.71 (*p* < 0.001)	3.22 (*p* = 0.0726)	8.25 (*p* = 0.0041)	-

Likelihood ratio test statistics based on first imputation. Complete case statistics available in supplementary material. *ICC* Intra-class correlation, *VPC* variance partition coefficient. Gender and family structure controlled for in all models

^*^Significant p-values indicate that the reduced model was a significantly worse fit than the three-level full model

**Table 4:** Multi-level logistic regression (behavioural difficulties) (*n* = 32,606)

	Model 1: Three level, no individual perceptions; no year group or school climate	Model 2: Three level with individual level climate, but no year group or school level climate (OR, 95%CI)	Model 3: Three level with year group climate only (OR, 95%CI)	Model 4: Three level with school climate only (OR, 95%CI)	Model 5 (Full model): Three level with year group and school climate (OR, 95%CI)
School year	1.08 (1.05–1.12) *p* < 0.001	0.99 (0.95–1.02) *p* = 0.467	0.95 (0.92–0.99) *p* = 0.014	0.99 (0.95–1.02) *p* = 0.472	0.96 (0.93–1.00) *p* = 0.064
Teachers care	-	0.82 (0.79–0.86) *p* < 0.001	0.83 (0.80–0.87) *p* < 0.001	0.83 (0.79–0.86) *p* < 0.001	0.83 (0.79–0.87) *p* < 0.001
Belong	-	0.80 (0.77–0.83) *p* < 0.001	0.81 (0.78–0.84) *p* < 0.001	0.80 (0.77–0.83) *p* < 0.001	0.81 (0.78–0.84) *p* < 0.001
Like school	-	0.64 (0.62–0.67) *p* < 0.001	0.65 (0.62–0.68) *p* < 0.001	0.65 (0.62–0.68) *p* < 0.001	0.65 (0.62–0.68) *p* < 0.001
Year group climate	-	-	0.87 (0.82–0.92) *p* < 0.001	-	0.91 (0.84–0.98) *p* = 0.010
School climate	-	-	-	0.81 (0.73–0.89) *p* < 0.001	0.89 (0.79–1.00) *p* = 0.055
Year group ICC (95%CI)	0.061 (0.047–0.074)	0.046 (0.034–0.058)	0.043 (0.031–0.055)	0.044 (0.032–0.056)	0.043 (0.031–0.055)
School ICC (95%CI)	0.032 (0.021–0.043)	0.024 (0.014–0.033)	0.021 (0.012–0.031)	0.021 (0.012–0.030)	0.021 (0.012–0.030)
Year group/ School VPC	2.85%/3.23%	2.22%/2.37%	2.20%/2.13%	2.27%/2.09%	2.20%/2.10%
Likelihood ratio test (compared to Model 5—full model)	1657.67 (*p* < 0.001)	23.94 (*p* < 0.001)	3.72 (*p* = 0.0539)	6.57 (*p* = 0.0104)	-

Likelihood ratio test statistics based on first imputation. Complete case statistics available in supplementary material. *ICC* Intra-class correlation, *VPC* variance partition coefficient. Gender and family structure controlled for in all models

^*^Significant *p*-values indicate that the reduced model was a significantly worse fit than the three-level full model

In all models, more positive individual perceptions of school climate (teachers care, were significantly associated with lower emotional and behavioural difficulties (*p* < 0.001 for all models). For emotional difficulties, perceptions of belonging had the strongest association (OR range = 0.68–0.69 across all models), while for behavioural difficulties, liking school had the strongest association (OR range = 0.64–0.65 across all models). Both indicate that higher levels of school climate at the individual level are associated with a reduction in the odds of having elevated or clinically significant difficulties of over 30% if all other factors are held constant.

On their own, more positive perceptions of year group and school climate measures were significantly associated with better mental health outcomes, and this direction of effect was retained when both measures were added into the full models. In the full models, year group and school climate measures had similar odds ratios (for emotional difficulties, 0.91 for year group climate (*p* = 0.004) and 0.92 for school climate (*p* = 0.092); for behavioural difficulties, 0.91 for year group climate (*p* = 0.010) and 0.89 for school climate (*p* = 0.055)), but only year group climate remained statistically significant (RQ2). This is likely to be due to lower power at the school level: while there were just 354 schools, there were 1,340 year group clusters, and this difference in sample size when the effect size is held constant, will result in greater power to detect effects at the year group level than at the school level.

## Discussion

This research has explored clustering of mental health outcomes at year group and school levels within children in their final four years of primary school in Wales. It uses ICCs (correlations between the mental health outcomes of different students based on the year group and school they attend) and VPCs (proportion of variability at different levels of the models – individual, year group, school) to explain the extent to which student mental health outcomes are related to the year group and school they attend, and the extent to which it is based on individual factors.

Estimates of unadjusted ICCs within two level models of children nested within schools were low, in line with those found in previous studies (Parker et al., 2023; Shackleton et al., 2016). Low year group and school ICCs (all < 0.07 across the different models), could either mean that schools have small effects on child mental health, or that schools have a larger effect, but that this effect is relatively universal across schools because schools are similar in the practices that harm or benefit mental health. This universality would result in more similarity between schools and year groups in child mental health status, and therefore less evidence of clustering. However, this research suggests that there remains sufficient variability at year group and school level to justify using three-level models. Some of the clustering effect at the year group level in the unadjusted models is likely to be attributable to an age group effect, highlighting that students in a particular year group are more alike each other in their mental health status than they are like students in a different year of the same school. It reinforces the importance of considering the needs of children of different ages and developmental stages within a school setting as they may require different pedagogical and relational approaches to support their mental health, and to create a climate that fosters their wellbeing. However, the analysis also found that even after controlling for school year, there remained evidence of year group clustering, and this may reflect effects that are contextual in nature (due to the school environment itself). It is therefore important to consider both compositional (including age) and contextual (including culture) factors when planning interventions or analyses within the primary school system at both year group and school level.

A consistent finding across all models was that more positive individual perceptions of school climate were significantly associated with more favourable mental health. This has been researched extensively in secondary schools with older children (Aldridge & McChesney, 2018) and our own research has shown that similar measures predict mental health outcomes in children in the first year of secondary school in Wales (Donaldson et al., 2024). However, this paper builds evidence of the importance of individual perceptions of school climate as predictors of mental health in primary-aged children. Furthermore, the research highlights the importance of both year group and school climate as aggregates of individual perceptions. Separately, both year group and school climate measures significantly predicted emotional and behavioural difficulties, and while together, only year group climate was significant, the odds ratios for both year group and school climate were very similar. Lack of significance for school climate may be largely a consequence of there being more units at the year group level and therefore more power. The odds ratios for the full model suggest that for emotional difficulties, one unit increase in either year group or school climate equates to an 8–9% reduction in the odds of emotional difficulties and a 9–11% reduction in the odds of behavioural difficulties, after adjusting for the other predictors. This could have a large impact on child outcomes as nearly 28% of children within this sample had elevated or clinically significant emotional difficulties and 14% had elevated or clinically significant behavioural difficulties (Appendix 1). Longitudinal analysis from UK secondary schools by Hinze et al. (2024) found school effects were important for explaining differences in child mental health and that school climate as perceived by children (both at the individual and aggregated school level) was the “single most important factor” associated with the mental health outcomes (p.279). The research presented in this paper goes further by arguing that year groups may also create their own climate, both due to compositional and contextual factors, that can further explain clustering of mental health at different levels of nesting within school.

Finally, even after all predictors have been added to the models, there remained evidence of clustering at both year group and school level, and the three level models remained a significantly better fit for the data than two or one level models. It is therefore of interest to consider what other factors, not included within this analysis, might explain some of the remaining variation. These are likely to be both compositional and contextual in nature (Bonell et al., 2013). Two key compositional factors that are likely to cluster in schools but were not included in this analysis are ethnicity (this was not collected in the survey) and socioeconomic status (not collected for younger children). Future iterations of the survey could look to include these questions to allow future exploration of these factors. There are also likely to be contextual factors that have not been accounted for, and the questions used in this analysis to assess school climate do not account for all dimensions of the construct that might influence child mental health, for example, aspects of the school physical environment, feelings of safety within school or peer relationships (Wang & Degol, 2016).

This research provides an important perspective on clustering of mental health outcomes within primary schools at three nested levels, using a large, representative sample of children and validated measures of mental health difficulties. It provides evidence that year group has important effects on child mental health and that school-based interventions for mental health need to consider both year group effects as well as school level effects. Focusing solely on school-wide approaches without considering how children may differ in terms of developmental needs at lower levels within the school system or how lower levels of nesting might creating their own climates that oppose overall school effects, may affect intervention effectiveness.

It does however have a number of limitations. The measures used to assess individual, year group and school climate were the same, aggregated at different levels, rather than specific questions about perceptions of each level. This may mean that the measures fail to capture aspects of climate that might be specific to the different levels, and future analysis should consider using scales that specifically focus on year group as well as school level perceptions. The study was cross sectional, and therefore it is not possible to assess the direction of association between mental health and year group/school climate. It is likely that the association is complex and not uni-directional – while school climate may be influencing mental health outcomes, it is also highly conceivable that schools with lower levels of mental health difficulties for reasons outside of the school context and outside of the variables controlled for within this study, will have better child perceptions of school climate. Longitudinal research to explore how school/year group climate and mental health change and interact over time would help build further understanding of their relationship. Prior to adjusting for child age within the models, year group effects cannot be separated from age group effects, and future work comparing year group effects with class effects where there are multiple classes in a year group, would help to disentangle the impacts of age from the impacts of school contextual effects. Future research may also wish to capture age rather than using school year as a proxy to account for any minor variations in age within the year group.. There may also be loss of power as a result of using binary outcome measures for the mental health measures. The findings here are situated within the Welsh educational system and therefore may not be generalisable to all other systems. Replication of the analyses in other educational contexts or regions, particularly alongside an understanding of school practices and policy, would provide an understanding of how different contexts might result in the same or different mechanisms involved in these effects being activated or deactivated.

The research also only focuses on child perceptions of year group and school climate, and future research should consider how teacher and other perspectives might help build a fuller picture of the relationship between year group/school climate and mental health (Grazia & Molinari, 2021). This is particularly important as using aggregates of individual-level reports to predict self-report outcomes for the same individuals may introduce same source bias into the analysis (Bonell et al., 2013). Furthermore, as assessments of year group/school climate within this study were necessarily based on self-report data from young children, it would help overcome any possible bias that mixed levels of comprehension or social desirability reporting may have introduced. There is also a need for a more consistent approach to measuring school climate across research, and this study was restricted to using those measures available within the survey. The measures used here may also be limited in their ability to capture differences between class and school climate as they ask for general perceptions without specifying between the environments. There was a small amount of missingness in the data and there was evidence that some of this was missing at random (MAR), which multiple imputation can help to address. However it is possible that there remained an element of data being missing not at random (MNAR) and this may have skewed findings in both the imputed and complete case analysis. Finally, the survey data collection period was over six months which may introduce temporal biases due to seasonal effects or school-term variation, and the use of an online survey may have led to difficulties accessing the survey for some respondents or schools.

## Conclusion

Child mental health outcomes cluster within year groups as well as within schools. Our findings are consistent with the presence of independent to year group and school effects, and makes a strong case for considering nesting at different levels within a school system when designing interventions to support mental health. Furthermore, there is evidence that individual perceptions of school climate predict mental health outcomes in primary-aged children, and that measures of year group and school climate provide evidence of a contextual effect on outcomes.

## Supplementary Information

Below is the link to the electronic supplementary material.

Supplementary file1 (DOCX 56 KB)
