# Supplementary material for: Associations of Perceived School and Year Group Climate with Mental Health Among Children Aged 7-to-11 Years
Source: Child Indic Res. 2025 Jan 8;18(2):885–904. doi: 10.1007/s12187-024-10213-7 (PMC11906510; doi:10.1007/s12187-024-10213-7)
Supplement: Supplementary file 1 — Supplementary file1 (DOCX 56 KB) [file 12187_2024_10213_MOESM1_ESM.docx]

**SUPPLEMENTARY MATERIAL**

**Appendix 1: Representativeness checks – data weighted by local authority and data without weighting.**

| Outcome | Weighted estimates (% or mean) | Unweighted estimates (% or mean) |
| --- | --- | --- |
|  |  |  |
| Emotional difficulties | 27.59% | 27.82% |
| Behavioural difficulties | 14.08% | 14.01% |
| Teachers care about me | 4.42 | 4.41 |
| I like school | 3.26 | 3.25 |
| I feel like I belong | 4.12 | 4.12 |

**Appendix 2. Frequency of number of classes within the sampled primary schools**

| Number of classes (across whole school)* | N (%) |
| --- | --- |
| 1 | 0 (0%) |
| 2 | 9 (2.6%) |
| 3 | 10 (2.9%) |
| 4 | 26 (7.6%) |
| 5 | 16 (4.7%) |
| 6 | 25 (7.3%) |
| 7 | 34 (9.9%) |
| 8 | 53 (15.5%) |
| 9 | 33 (9.7%) |
| 10 | 21 (6.1%) |
| 11 | 19 (5.6%) |
| 12 | 7 (2.1%) |
| 13 | 11 (3.2%) |
| 14 | 16 (4.7%) |
| 15 | 10 (2.9%) |
| 16 | 27 (7.9%) |
| 17 | 5 (1.5%) |
| 18 | 6 (1.8%) |
| 19 | 3 (0.9%) |
| 20 | 2 (0.6%) |
| 21 | 3 (0.9%) |
| 22 | 0 (0.0%) |
| 23 | 2 (0.6%) |
| 24 | 2 (0.6%) |
| 25 | 0 (0.0%) |
| 26 | 1 (0.3%) |
| 27 | 1 (0.3%) |
| Total schools with available data | 342 (100%) |

*Data not available for all-age schools

**Appendix 3: Questions used to calculate climate measures**

1. Thinking about the adults in your school, how much do you agree or disagree with the following sentences…?

- My teachers care about me as a person

Strongly agree; agree; neither agree nor disagree; disagree; strongly disagree; I do not want to answer*.

1. Thinking about your school, how much do you agree or disagree with the following sentences…?

- I feel like I belong at this school

Strongly agree; agree; neither agree nor disagree; disagree; strongly disagree; I do not want to answer*.

1. How do you feel about school?

I like it a lot; I like it a bit; I don’t like it very much; I don’t like it at all; I do not want to answer*.

*All ‘I do not want to answer’ responses were coded as missing

**Appendix 4: Correlations between school connectedness items at individual, year group and school level.**

|  | Individual (alpha = 0.69) | | | Year group (alpha = 0.83) | | | School (alpha = 0.86) | | |
| --- | --- | --- | --- | --- | --- | --- | --- | --- | --- |
|  | Teachers care | Belong | Like school | Teachers care | Belong | Like school | Teachers care | Belong | Like school |
| Teachers care | 1.0 |  |  | 1.0 |  |  | 1.0 |  |  |
| Belong | 0.47 | 1.0 |  | 0.66 | 1.0 |  | 0.72 | 1.0 |  |
| Like school | 0.36 | 0.48 | 1.0 | 0.56 | 0.67 | 1.0 | 0.62 | 0.71 | 1.0 |

Correlations are low (<0.50) for the individual items, and alpha is also below the cut off generally accepted as ‘good’ (<0.70). Therefore the individual items will be treated separately, and the year group/school items combined to form year group and school climate measures respectively.

**Appendix 5: Missingness in outcome and predictor variables. Total sample = 32,606.**

| **Variables** | **Missingness** |
| --- | --- |
|  |  |
| Emotional difficulties | 719 (2.2%) |
| Behavioural difficulties | 891 (2.7%) |
| Gender | 632 (1.9%) |
| Family structure | 1890 (5.8%) |
| Teachers care | 1928 (5.9%) |
| Belong | 2730 (8.4%) |
| Like school | 1860 (5.7%) |
| School year | 0 (0.0%) |

**Appendix 6: Variables selected for use in multiple imputation, and univariate analysis of their relationship with missingness in the two outcome variables (chi squared statistic and p-values).**

|  | Missingness in emotional difficulties | Missingness in behavioural difficulties |
| --- | --- | --- |
| Home language | 2.60 (p=0.458) | 6.12 (p=0.106) |
| Survey language | 0.53 (p=0.467) | 3.79 (p=0.051) |
| Gender | 5.95 (p=0.051) | 5.45 (p=0.065) |
| School year | 11.78 (p=0.008) | 23.97 (p<0.001) |
| Family structure | 3.54 (p=0.060) | 4.77 (p=0.029) |
| School affluence | 7.48 (p=0.024) | 5.47 (p=0.065) |
| Teachers care | 38.01 (p<0.001) | 40.20 (p<0.001) |
| Belong | 24.90 (p<0.001) | 38.08 (p<0.001) |
| Like school | 86.26 (p<0.001) | 82.52 (p<0.001) |
| Life satisfaction | 36.59 (p<0.001) | 52.22 (p<0.001) |
| Exercise | 4.37 (p=0.037) | 4.02 (p=0.045) |
| Bullying victimisation | 2.00 (p=0.158) | 4.13 (p=0.042) |

All selected variables were significantly associated with the outcome variables themselves, based on univariate analyses, except for survey language and behavioural outcomes (p=0.663).

Home language = language spoken at home by the respondent. Categories include English, Welsh, English and Welsh, or Other. Survey language = respondents could complete a Welsh or English language survey. School affluence = based on school percentage free school meal eligibility. Higher levels of %FSM indicate lower levels of school affluence. Life satisfaction = based on the Cantril ladder. Respondents were asked to select how they would rate their life at the moment from 0 (“I have the worst possible life) to 10 (“I have the best possible life”). Exercise = children selected how frequently they participated in exercise in a week. This was dichotomised into four or more times a week, and less than four times a week. Bullying victimisation = children selected whether they had been bullied at school in the past couple of months.

**Appendix 7: Sensitivity analysis 1: Complete case analysis. Intraclass correlations for emotional and behavioural difficulties (unadjusted and adjusted for all individual-level variables).**

|  | Three levels  (children in year groups in schools) | | Two level  (children in year groups) | | Two level  (children in schools) | |
| --- | --- | --- | --- | --- | --- | --- |
|  | Year group ICC  (95%CI)  VPC | School ICC  (95%CI)  VPC | Year group ICC  (95%CI)  VPC | Likelihood ratio test (compared to three level model) | School ICC  (95%CI)  VPC | Likelihood ratio test (compared to three level model) |
| Emotional difficulties unadjusted  (n=31,887) | 0.052  (0.043-0.062)  2.79% | 0.024  (0.017-0.033)  2.39% | 0.051  (0.043-0.061)  5.09% | 60.25  (p<0.001) | 0.031  (0.024-0.040)  3.12% | 79.74  (p<0.001) |
| Emotional difficulties adjusted  (school year)  (n=31,887) | 0.050  (0.042-0.061)  2.58% | 0.025  (0.018-0.034)  2.46% | 0.049  (0.041-0.059)  4.91% | 64.84  (p<0.001) | 0.031  (0.025-0.040)  3.13% | 69.51  (p<0.001) |
| Emotional difficulties adjusted  (school year, gender, family structure)  (n=29,740) | 0.049  (0.040-0.060)  2.48% | 0.024  (0.017-0.034)  2.42% | 0.047  (0.039-0.058)  4.73% | 57.60  (p<0.001) | 0.031  (0.024-0.040)  3.06% | 54.79  (p<0.001) |
|  |  |  |  |  |  |  |
| Behavioural difficulties unadjusted  (n=31,715) | 0.066  (0.054-0.081)  3.15% | 0.035  (0.025-0.048)  3.47% | 0.065  (0.053-0.079)  6.48% | 60.77  p<0.001 | 0.043  (0.033-0.055)  4.31% | 43.41  p<0.001 |
| Behavioural difficulties adjusted  (school year)  (n=31,715) | 0.064  (0.052-0.079)  2.82% | 0.036  (0.026-0.049)  3.58% | 0.063  (0.051-0.077)  6.25% | 65.82  p<0.001 | 0.043  (0.034-0.056)  4.33% | 35.47  p<0.001 |
| Behavioural difficulties adjusted  (school year, gender, family structure)  (n=29,598) | 0.060  (0.048-0.076)  2.71% | 0.033  (0.023-0.047)  3.31% | 0.059  (0.047-0.073)  5.87% | 52.26  p<0.001 | 0.040  (0.031-0.053)  4.03% | 27.30  p<0.001 |

*Significant p-values indicate that the reduced model was a significantly worse fit than the three-level model.

**All two-level models were significantly better fit to the data than a one-level model with no clustering effect (p<0.001 for all models).

Intra-class correlation (ICC); variance partition coefficient (VPC).

**Appendix 8: Sensitivity analysis 1: Complete case analysis. Multi-level logistic regression (emotional difficulties). All models adjusted for gender and family structure.**

|  | Model 1: Three level, no individual perceptions; no year group or school climate  (OR, 95%CI)  (n=29,740) | Model 2: Three level with no year group or school climate  (OR, 95%CI)  (n=26,136) | Model 3: Three level with year group climate only  (OR, 95%CI)  (n=26,136) | Model 4: Three level with school climate only  (OR, 95%CI)  (n=26,136) | Model 5 (Full model): Three level with year group and school climate  (OR, 95%CI)  (n=26,136) |
| --- | --- | --- | --- | --- | --- |
| School year | 1.07 (1.04-1.10)  p<0.001 | 0.98 (0.95-1.01)  p=0.188 | 0.95 (0.92-0.98)  p=0.004 | 0.98 (0.95-1.01)  p=0.186 | 0.96 (0.93-0.99)  p=0.020 |
|  |  |  |  |  |  |
| Teachers care | - | 0.87 (0.83-0.90)  p<0.001 | 0.87 (0.84-0.91)  p<0.001 | 0.87 (0.83-0.91)  p<0.001 | 0.87 (0.84-0.91)  p<0.001 |
| Belong | - | 0.67 (0.64-0.69)  p<0.001 | 0.67 (0.65-0.70)  p<0.001 | 0.67 (0.65-0.69)  p<0.001 | 0.67 (0.65-0.70)  p<0.001 |
| Like school | - | 0.72 (0.69-0.75)  p<0.001 | 0.73 (0.70-0.76)  p<0.001 | 0.73 (0.70-0.76)  p<0.001 | 0.73 (0.70-0.76)  p<0.001 |
|  |  |  |  |  |  |
| Year group climate | - | - | 0.89 (0.84-0.94)  p<0.001 | - | 0.92 (0.86-0.98)  p=0.014 |
| School climate | - | - | - | 0.84 (0.77-0.92)  p<0.001 | 0.91 (0.82-1.01)  p=0.079 |
|  |  |  |  |  |  |
| Year group ICC (95%CI) | 0.049  (0.040-0.060) | 0.039  (0.030-0.051) | 0.037  (0.028-0.049) | 0.037  (0.029-0.049) | 0.037  (0.028-0.048) |
| School ICC (95%CI) | 0.024  (0.017-0.034) | 0.017  (0.011-0.027) | 0.016  (0.010-0.025) | 0.015  (0.009-0.024) | 0.015  (0.009-0.025) |
| Year group/ School VPC | 2.48%/2.42% | 2.22%/1.73% | 2.17%/1.55% | 2.26%/1.48% | 2.18%/1.51% |
| Likelihood ratio test (compared to Model 5 - full model) | -** | 21.19  p<0.001 | 3.08  p=0.0793 | 6.00  p=0.0143 | - |

*Significant p-values indicate that the reduced model was a significantly worse fit than the full model.**Not possible to run likelihood ratio test as sample size differs between model 1 and model 5. Intra-class correlation (ICC); variance partition coefficient (VPC). Gender and family structure controlled for in all models.

**Appendix 9: Sensitivity analysis 1: Complete case analysis. Multi-level logistic regression (behavioural difficulties). All models adjusted for gender and family structure.**

|  | Model 1: Three level, no individual perceptions; no year group or school climate  (n=29,598) | Model 2: Three level with individual level climate, but no year group or school level climate  (OR, 95%CI)  (n=26,046) | Model 3: Three level with year group climate only  (OR, 95%CI)  (n=26,046) | Model 4: Three level with school climate only  (OR, 95%CI)  (n=26,046) | Model 5 (Full model): Three level with year group and school climate  (OR, 95%CI)  (n=26,046) |
| --- | --- | --- | --- | --- | --- |
| School year | 1.08 (1.05-1.12)  p<0.001 | 0.98 (0.94-1.02)  p=0.260 | 0.94 (0.90-0.98)  p=0.006 | 0.98 (0.94-1.02)  p=0.253 | 0.95 (0.91-1.00)  p=0.030 |
| Teachers care | - | 0.81 (0.77-0.85)  p<0.001 | 0.82 (0.78-0.86)  p<0.001 | 0.81 (0.78-0.86)  p<0.001 | 0.82 (0.78-0.86)  p<0.001 |
| Belong | - | 0.79 (0.76-0.82)  p<0.001 | 0.80 (0.76-0.83)  p<0.001 | 0.79 (0.76-0.83)  p<0.001 | 0.80 (0.76-0.83)  p<0.001 |
| Like school | - | 0.62 (0.59-0.65)  p<0.001 | 0.63 (0.60-0.66)  p<0.001 | 0.63 (0.60-0.66)  p<0.001 | 0.63 (0.60-0.66)  p<0.001 |
| Year group climate | - | - | 0.86 (0.80-0.92)  p<0.001 | - | 0.90 (0.83-0.98)  p=0.012 |
| School climate | - | - | - | 0.80 (0.73-0.89)  p<0.001 | 0.90 (0.78-1.01)  p=0.071 |
|  |  |  |  |  |  |
| Year group ICC  (95%CI) | 0.060  (0.048-0.076) | 0.046  (0.033-0.063) | 0.043  (0.031-0.060) | 0.044  (0.031-0.060) | 0.043  (0.030-0.059) |
| School ICC  (95%CI) | 0.033  (0.023-0.047) | 0.022  (0.013-0.036) | 0.019  (0.011-0.033) | 0.018  (0.010-0.033) | 0.018  (0.010-0.033) |
| Year group/ School VPC | 2.71%/3.31% | 2.44%/2.16% | 2.40%/1.88% | 2.53%/1.83% | 2.41%/1.84% |
| Likelihood ratio test (compared to Model 5 - full model) | -** | 22.79  p<0.001 | 3.24  p=0.0717 | 6.30  p=0.0120 | - |

*Significant p-values indicate that the reduced model was a significantly worse fit than the full model. **Not possible to run likelihood ratio test as sample size differs between model 1 and model 5. Intra-class correlation (ICC); variance partition coefficient (VPC). Gender and family structure controlled for in all models.

**Appendix 10: Sensitivity analysis 2: Two-level substantive models using two-level imputation models; three-level model using three-level imputation model. Intraclass correlations for emotional and behavioural difficulties (unadjusted and adjusted for all individual-level variables) (n=32,606).**

|  | Three levels  (children in year groups in schools) | | Two level  (children in year groups) | | Two level  (children in schools) | |
| --- | --- | --- | --- | --- | --- | --- |
|  | Year group ICC  (95%CI)  VPC | School ICC  (95%CI)  VPC | Year group ICC  (95%CI)  VPC | Likelihood ratio test (compared to three level model) | School ICC  (95%CI)  VPC | Likelihood ratio test (compared to three level model) |
| Emotional difficulties unadjusted | 0.052  (0.042-0.061)  2.81% | 0.024  (0.016-0.032)  2.37% | 0.050  (0.042-0.059)  5.04% | 116.44  (p<0.001) | 0.031  (0.023-0.038)  3.09% | 109.20  (p<0.001) |
| Emotional difficulties adjusted (school year) | 0.050  (0.041-0.060)  2.60% | 0.024  (0.016-0.032)  2.44% | 0.049  (0.040-0.058)  4.87% | 122.03  (p<0.001) | 0.031  (0.024-0.039)  3.10% | 97.73  (p<0.001) |
| Emotional difficulties adjusted  (school year, gender, family structure) | 0.049  (0.040-0.059)  2.58% | 0.023  (0.016-0.031)  2.34% | 0.047  (0.039-0.056)  4.75% | 137.45  (p<0.001) | 0.030  (0.023-0.038)  3.01% | 108.04  (p<0.001) |
|  |  |  |  |  |  |  |
| Behavioural difficulties  unadjusted | 0.067  (0.053-0.080)  3.20% | 0.035  (0.023-0.046)  3.47% | 0.065  (0.053-0.078)  6.53% | 28.14  (p<0.001) | 0.043  (0.032-0.054)  4.28% | 78.03  (p<0.001) |
| Behavioural difficulties adjusted  (school year) | 0.064  (0.051-0.078)  2.87% | 0.036  (0.024-0.047)  3.57% | 0.063  (0.050-0.076)  6.31% | 34.24  (p<0.001) | 0.043  (0.032-0.054)  4.30% | 68.90  (p<0.001) |
| Behavioural difficulties adjusted  (school year, gender, family structure) | 0.061  (0.047-0.074)  2.85% | 0.032  (0.021-0.043)  3.23% | 0.059  (0.047-0.072)  5.92% | 13.21  (p<0.001) | 0.039  (0.029-0.050)  3.93% | 71.58  (p<0.001) |

*Significant p-values indicate that the reduced model was a significantly worse fit than the three-level model. **All two-level models were significantly better fit to the data than a one-level model with no clustering effect (p<0.001 for all models).

Likelihood ratio test statistics based on first imputation. Complete case statistics available in supplementary material. Intra-class correlation (ICC); variance partition coefficient (VPC).

**Appendix 11: Sensitivity analysis 3: Missing Not At Random (MNAR) sensitivity analysis. Intraclass correlations for emotional and behavioural difficulties (unadjusted and adjusted for all individual-level variables) (n=32,606). All missing values of the outcome variable have been replaced with ‘0’ across the imputed datasets.**

|  | Three levels  (children in year groups in schools) | | Two level  (children in year groups) | | Two level  (children in schools) | |
| --- | --- | --- | --- | --- | --- | --- |
|  | Year group ICC  (95%CI)  VPC | School ICC  (95%CI)  VPC | Year group ICC  (95%CI)  VPC | Likelihood ratio test (compared to three level model) | School ICC  (95%CI)  VPC | Likelihood ratio test (compared to three level model) |
| Emotional difficulties unadjusted | 0.049 (0.040-0.058)  2.68% | 0.022 (0.015-0.030)  2.24% | 0.048 (0.039-0.057)  4.82% | 56.33  p<0.001 | 0.029 (0.022-0.037)  2.94% | 75.48  p<0.001 |
| Emotional difficulties adjusted  (school year) | 0.047 (0.038-0.057)  2.44% | 0.023 (0.015-0.031)  2.31% | 0.046 (0.038-0.055)  4.63% | 61.29  p<0.001 | 0.029 (0.022-0.037)  2.94% | 64.37  p<0.001 |
| Emotional difficulties adjusted  (school year, gender, family structure) | 0.046 (0.037-0.056)  2.43% | 0.022 (0.015-0.030)  2.22% | 0.045 (0.036-0.054)  4.51% | 56.07  p<0.001 | 0.029 (0.021-0.036)  2.86% | 62.12  p<0.001 |
|  |  |  |  |  |  |  |
| Behavioural difficulties unadjusted | 0.065 (0.051-0.078)  3.11% | 0.034 (0.022-0.045)  3.36% | 0.063 (0.051-0.076)  6.33% | 58.66  p<0.001 | 0.042 (0.031-0.053)  4.18% | 43.49  p<0.001 |
| Behavioural difficulties adjusted  (school year) | 0.062 (0.049-0.076)  2.75% | 0.035 (0.024-0.046)  3.47% | 0.061 (0.048-0.073)  6.08% | 64.22  p<0.001 | 0.042 (0.031-0.053)  4.21% | 34.56  p<0.001 |
| Behavioural difficulties adjusted  (school year, gender, family structure) | 0.058 (0.045-0.072)  2.73% | 0.031 (0.020-0.042)  3.12% | 0.057 (0.045-0.069)  5.70% | 53.48  p<0.001 | 0.038 (0.028-0.049)  3.84% | 33.11  p<0.001 |

*Significant p-values indicate that the reduced model was a significantly worse fit than the three-level model.

**All two-level models were significantly better fit to the data than a one-level model with no clustering effect (p<0.001 for all models).

Intra-class correlation (ICC); variance partition coefficient (VPC).

**Appendix 12: Sensitivity analysis 3: Missing Not At Random (MNAR) sensitivity analysis. Multi-level logistic regression (emotional difficulties) (n=32,606). All missing values of the outcome variable have been replaced with ‘0’ across the imputed datasets.**

|  | Model 1: Three level, no individual perceptions; no year group or school climate  (OR, 95%CI) | Model 2: Three level with no year group or school climate  (OR, 95%CI) | Model 3: Three level with year group climate only  (OR, 95%CI) | Model 4: Three level with school climate only  (OR, 95%CI) | Model 5 (Full model): Three level with year group and school climate  (OR, 95%CI) |
| --- | --- | --- | --- | --- | --- |
| School year | 1.07 (1.05-1.10)  p<0.001 | 0.99 (0.97-1.02)  p=0.580 | 0.97 (0.94-0.99)  p=0.021 | 0.99 (0.97-1.02) p=0.586 | 0.97 (0.94-1.00)  p=0.075 |
|  |  |  |  |  |  |
| Teachers care | - | 0.88 (0.85-0.92)  p<0.001 | 0.89 (0.86-0.92)  p<0.001 | 0.89 (0.85-0.92)  p<0.001 | 0.89 (0.86-0.92)  p<0.001 |
| Belong | - | 0.69 (0.67-0.71)  p<0.001 | 0.69 (0.67-0.71)  p<0.001 | 0.69 (0.67-0.71)  p<0.001 | 0.69 (0.67-0.71)  p<0.001 |
| Like school | - | 0.74 (0.71-0.76)  p<0.001 | 0.74 (0.72-0.77)  p<0.001 | 0.74 (0.72-0.77)  p<0.001 | 0.74 (0.72-0.77)  p<0.001 |
|  |  |  |  |  |  |
| Year group climate | - | - | 0.89 (0.85-0.94)  p<0.001 | - | 0.92 (0.87-0.98)  p=0.006 |
| School climate | - | - | - | 0.85 (0.79-0.92)  p<0.001 | 0.92 (0.84-1.02)  p=0.101 |
|  |  |  |  |  |  |
| Year group ICC (95%CI) | 0.046  (0.037-0.056) | 0.034  (0.026-0.042) | 0.032  (0.024-0.040) | 0.032  (0.024-0.041) | 0.032  (0.024-0.040) |
| School ICC (95%CI) | 0.022  (0.015-0.030) | 0.016  (0.009-0.022) | 0.015  (0.008-0.021) | 0.014  (0.008-0.020) | 0.014  (0.008-0.021) |
| Year group/ School VPC | 2.43%/2.22% | 1.81%/1.59% | 1.76%/1.46% | 1.83%/1.41% | 1.76%/1.43% |
| Likelihood ratio test (compared to Model 5 - full model) | 2263.11  p<0.001 | 24.47  p<0.001 | 2.34  p=0.1259 | 8.89  p=0.0029 | - |

*Significant p-values indicate that the reduced model was a significantly worse fit than the three-level model. Likelihood ratio test statistics based on first imputation. Intra-class correlation (ICC); variance partition coefficient (VPC). Gender and family structure controlled for in all models.

**Appendix 13: Sensitivity analysis 3: Missing Not At Random (MNAR) sensitivity analysis. Logistic regression (behavioural difficulties) (n=32,606). All missing values of the outcome variable have been replaced with ‘0’ across the imputed datasets.**

|  | Model 1: Three level, no individual perceptions; no year group or school climate  (OR, 95%CI) | Model 2: Three level with individual level climate, but no year group or school level climate  (OR, 95%CI) | Model 3: Three level with year group climate only  (OR, 95%CI) | Model 4: Three level with school climate only  (OR, 95%CI) | Model 5 (Full model): Three level with year group and school climate  (OR, 95%CI) |
| --- | --- | --- | --- | --- | --- |
| School year | 1.09 (1.05-1.13)  p<0.001 | 1.00 (0.96-1.03)  p=0.817 | 0.96 (0.93-1.00)  p=0.048 | 1.00 (0.96-1.03)  p=0.822 | 0.97 (0.94-1.01)  p=0.166 |
| Teachers care | - | 0.83 (0.79-0.87)  p<0.001 | 0.84 (0.80-0.88)  p<0.001 | 0.83 (0.80-0.87)  p<0.001 | 0.84 (0.80-0.88)  p<0.001 |
| Belong | - | 0.81 (0.78-0.84)  p<0.001 | 0.81 (0.78-0.84)  p<0.001 | 0.81 (0.78-0.84)  p<0.001 | 0.81 (0.78-0.84)  p<0.001 |
| Like school | - | 0.65 (0.63-0.68)  p<0.001 | 0.66 (0.63-0.69)  p<0.001 | 0.66 (0.63-0.69)  p<0.001 | 0.66 (0.64-0.69)  p<0.001 |
|  |  |  |  |  |  |
| Year group climate | - | - | 0.87 (0.82-0.93)  p<0.001 | - | 0.91 (0.85-0.98)  p=0.011 |
| School climate | - | - | - | 0.81 (0.74-0.90)  p<0.001 | 0.89 (0.79-1.00)  p=0.049 |
|  |  |  |  |  |  |
| Year group ICC  (95%CI) | 0.058  (0.045-0.072) | 0.044  (0.032-0.056) | 0.042  (0.030-0.053) | 0.042  (0.030-0.054) | 0.041  (0.030-0.053) |
| School ICC  (95%CI) | 0.031  (0.020-0.042) | 0.023  (0.014-0.033) | 0.021  (0.012-0.030) | 0.020  (0.011-0.030) | 0.021  (0.011-0.030) |
| Year group/ School VPC | 2.73%/3.12% | 2.11%/2.31% | 2.07%/2.08% | 2.15%/2.05% | 2.07%/2.06% |
| Likelihood ratio test (compared to Model 5 - full model) | 1486.99  p<0.001 | 25.05  p<0.001 | 3.63  p=0.0567 | 7.31  p=0.0069 | - |

*Significant p-values indicate that the reduced model was a significantly worse fit than the three-level model.

Likelihood ratio test statistics based on first imputation. Intra-class correlation (ICC); variance partition coefficient (VPC). Gender and family structure controlled for in all models.

**Appendix 14: Sensitivity analysis 4: Excluding schools with 4 or fewer classes (Complete case analysis). Intraclass correlations for emotional and behavioural difficulties (unadjusted and adjusted for all individual-level variables). [This was to check whether schools with low class numbers were skewing results due to low variability at this level]**

|  | | Three levels  (children in year groups in schools) | | Two level  (children in year groups) | | Two level  (children in schools) | |  |
| --- | --- | --- | --- | --- | --- | --- | --- | --- |
|  | | Year group ICC  (95%CI)  VPC | School ICC  (95%CI)  VPC | Year group ICC  (95%CI)  VPC | Likelihood ratio test (compared to three level model) | School ICC  (95%CI)  VPC | Likelihood ratio test (compared to three level model) |  |
| Emotional difficulties unadjusted  (n=30,454) | | 0.051  (0.042-0.061)  2.78% | 0.023  (0.016-0.032)  2.29% | 0.050  (0.042-0.060)  5.01% | 55.39  p<0.001 | 0.030  (0.024-0.039)  3.02% | 77.82  p<0.001 |  |
| Emotional difficulties adjusted  (school year)  (n=30,454) | | 0.049  (0.040-0.060)  2.57% | 0.023  (0.017-0.033)  2.35% | 0.048  (0.040-0.058)  4.84% | 59.41  p<0.001 | 0.030  (0.024-0.039)  3.02% | 67.99  p<0.001 |  |
| Emotional difficulties adjusted  (school year, gender, family structure)  (n=28,377) | | 0.048  (0.039-0.059)  2.48% | 0.023  (0.016-0.033)  2.31% | 0.047  (0.038-0.057)  4.65% | 52.65  p<0.001 | 0.029  (0.023-0.038)  2.95% | 53.68  p<0.001 |  |
|  | |  |  |  |  |  |  |  |
| Behavioural difficulties unadjusted  (n=30,287) | 0.064  (0.052-0.079)  3.16% | | 0.033  (0.023-0.046)  3.26% | 0.064  (0.052-0.078)  6.39% | 54.68  p<0.001 | 0.041  (0.032-0.053)  4.11% | 42.84  p<0.001 | |
| Behavioural difficulties adjusted  (school year)  (n=30,287) | 0.062  (0.050-0.077)  2.84% | | 0.034  (0.024-0.047)  3.37% | 0.062  (0.050-0.076)  6.17% | 59.36  p<0.001 | 0.041  (0.032-0.054)  4.13% | 35.11  p<0.001 | |
| Behavioural difficulties adjusted  (school year, gender, family structure)  (n=28,240) | 0.059  (0.046-0.074)  2.75% | | 0.031  (0.022-0.045)  3.11% | 0.058  (0.046-0.073)  5.79% | 46.72  p<0.001 | 0.038  (0.029-0.051)  3.84% | 27.35  p<0.001 | |

*Significant p-values indicate that the reduced model was a significantly worse fit than the three-level model. **All two-level models were significantly better fit to the data than a one-level model with no clustering effect (p<0.001 for all models). Intra-class correlation (ICC); variance partition coefficient (VPC).

**Appendix 15: Sensitivity analysis 4: Excluding schools with 4 or fewer classes (Complete case analysis). Multi-level logistic regression (emotional difficulties). [This was to check whether schools with low class numbers were skewing results due to low variability at this level]**

|  | Model 1: Three level, no individual perceptions; no year group or school climate  (OR, 95%CI)  (n=28,377) | Model 2: Three level with no year group or school climate  (OR, 95%CI)  (n=24,918) | Model 3: Three level with year group climate only  (OR, 95%CI)  (n=24,918) | Model 4: Three level with school climate only  (OR, 95%CI)  (n=24,918) | Model 5 (Full model): Three level with year group and school climate  (OR, 95%CI)  (n=24,918) |
| --- | --- | --- | --- | --- | --- |
| School year | 1.07 (1.04-1.10)  p<0.001 | 0.98 (0.95-1.01)  p=0.175 | 0.95 (0.92-0.98)  p=0.003 | 0.98 (0.95-1.01) p=0.169 | 0.96 (0.92-0.99)  p=0.017 |
|  |  |  |  |  |  |
| Teachers care | - | 0.86 (0.83-0.90)  p<0.001 | 0.87 (0.83-0.91)  p<0.001 | 0.87 (0.83-0.90)  p<0.001 | 0.87 (0.83-0.91)  p<0.001 |
| Belong | - | 0.67 (0.64-0.69)  p<0.001 | 0.67 (0.65-0.69)  p<0.001 | 0.67 (0.65-0.69)  p<0.001 | 0.67 (0.65-0.69)  p<0.001 |
| Like school | - | 0.72 (0.69-0.75)  p<0.001 | 0.73 (0.70-0.76)  p<0.001 | 0.73 (0.70-0.76)  p<0.001 | 0.73 (0.70-0.76)  p<0.001 |
|  |  |  |  |  |  |
| Year group climate | - | - | 0.88 (0.83-0.94)  p<0.001 | - | 0.91 (0.85-0.98)  p=0.013 |
| School climate | - | - | - | 0.84 (0.76-0.92)  p<0.001 | 0.91 (0.81-1.02)  p=0.100 |
|  |  |  |  |  |  |
| Year group ICC (95%CI) | 0.048  (0.039-0.059) | 0.039  (0.030-0.051) | 0.037  (0.028-0.049) | 0.037  (0.028-0.049) | 0.037  (0.028-0.048) |
| School ICC (95%CI) | 0.023  (0.016-0.033) | 0.017  (0.011-0.027) | 0.016  (0.010-0.025) | 0.015  (0.009-0.025) | 0.015  (0.009-0.025) |
| Year group/ School VPC | 2.48%/2.31% | 2.19%/1.75% | 2.14%/1.57% | 2.22%/1.50% | 2.15%/1.53% |
| Likelihood ratio test (compared to Model 5 - full model) | *Can’t run as sample size differs to full model | 20.17  p<0.001 | 2.69  p=0.1009 | 6.10  p=0.0135 | - |

*Significant p-values indicate that the reduced model was a significantly worse fit than the three-level model.

Intra-class correlation (ICC); variance partition coefficient (VPC). Gender and family structure controlled for in all models.

**Appendix 16: Sensitivity analysis 4: Excluding schools with 4 or fewer classes (Complete case analysis). Multi-level logistic regression (behavioural difficulties). [This was to check whether schools with low class numbers were skewing results due to low variability at this level]**

|  | Model 1: Three level, no individual perceptions; no year group or school climate  (OR, 95%CI)  (n=28,240) | Model 2: Three level with individual level climate, but no year group or school level climate  (OR, 95%CI)  (n=24,830) | Model 3: Three level with year group climate only  (OR, 95%CI)  (n=24,830) | Model 4: Three level with school climate only  (OR, 95%CI)  (n=24,830) | Model 5 (Full model): Three level with year group and school climate  (OR, 95%CI)  (n=24,830) |
| --- | --- | --- | --- | --- | --- |
| School year | 1.08 (1.04-1.12)  p<0.001 | 0.98 (0.94-1.02)  p=0.270 | 0.94 (0.90-0.99)  p=0.009 | 0.98 (0.94-1.02)  p=0.259 | 0.95 (0.91-1.00)  p=0.036 |
| Teachers care | - | 0.81 (0.77-0.85)  p<0.001 | 0.82 (0.78-0.86)  p<0.001 | 0.81 (0.77-0.85)  p<0.001 | 0.82 (0.78-0.86)  p<0.001 |
| Belong | - | 0.79 (0.76-0.82)  p<0.001 | 0.79 (0.76-0.83)  p<0.001 | 0.79 (0.76-0.82)  p<0.001 | 0.79 (0.76-0.83)  p<0.001 |
| Like school | - | 0.62 (0.59-0.65)  p<0.001 | 0.63 (0.60-0.66)  p<0.001 | 0.63 (0.60-0.66)  p<0.001 | 0.63 (0.60-0.66)  p<0.001 |
|  |  |  |  |  |  |
| Year group climate | - | - | 0.87 (0.81-0.93)  p<0.001 | - | 0.90 (0.83-0.98)  p=0.019 |
| School climate | - | - | - | 0.82 (0.73-0.91)  p<0.001 | 0.90 (0.79-1.03)  p=0.135 |
|  |  |  |  |  |  |
| Year group ICC  (95%CI) | 0.059  (0.046-0.074) | 0.044  (0.032-0.061) | 0.041  (0.029-0.058) | 0.042  (0.030-0.059) | 0.041  (0.029-0.058) |
| School ICC  (95%CI) | 0.031  (0.022-0.045) | 0.021  (0.012-0.035) | 0.018  (0.010-0.032) | 0.018  (0.010-0.032) | 0.018  (0.010-0.032) |
| Year group/ School VPC | 2.75%/3.11% | 2.38%/2.05% | 2.33%/1.81% | 2.45%/1.75% | 2.34%/1.77% |
| Likelihood ratio test (compared to Model 5 - full model) | *Can’t run as sample size differs to full model | 18.17  p<0.001 | 2.23  p=0.1357 | 5.45  p=0.0196 | - |

*Significant p-values indicate that the reduced model was a significantly worse fit than the three-level model.

Intra-class correlation (ICC); variance partition coefficient (VPC). Gender and family structure controlled for in all models.
